# The genesis, development and implementation of an interdisciplinary university Cross-School Research Group

**DOI:** 10.1007/s13384-022-00513-8

**Published:** 2022-03-29

**Authors:** Robyn Brandenburg, Jeremy Smith, Angela Higgins, Jerry Courvisanos

**Affiliations:** 1 School of Education, Federation University Australia, Ballarat, Australia; 2 School of Arts, Federation University Australia, Ballarat, Australia; 3 Federation Business School, Federation University Australia, Ballarat, Australia

**Keywords:** Interdisciplinary research, Collaboration, Collegiality, Habermas theory

## Abstract

This article examines the genesis, development and implementation of an interdisciplinary university cross-school research group (three individual schools) at Federation University in Australia. This CSRG is a consequence of both local and national calls for interdisciplinarity in university research and a direct response to the revised Strategic Goals and Policy document at Federation University. Using a conceptual framework based on a treatise by Jürgen Habermas (The theory of communicative action, Beacon Press, 1987) incorporating three socio-political levels (Lifeworld, Steering Media and Systems), we examined the ideals, processes and challenges in setting up an interdisciplinary research group within a traditional disciplinary-based university environment. Drawing on multiple data sets composed of member survey responses and interviews, email communication, online meetings, policy documents and co-leader feedback, we identified key resonant themes focussing on academic aspiration and motivation, the role of policy and practice, influence of grants and grant development across schools, mentoring and publishing. Using Habermas’ conceptual framework and his overarching notion of Lifeworld with qualitative methods of data analysis, this article explores establishment of the CSRG, deeper academic aspirations and engagement for interdisciplinarity informing the group’s formation and effectiveness of the processes used in this specific case. The impact on systems and policy is addressed together with the processes adopted to bring about interdisciplinary university collaboration. Evaluating the formation of the CSRG, the authors found that researchers placed a high value on opportunities to creatively collaborate in a cross-school and interdisciplinary environment, whereas obtaining grants and publishing research were seen by staff as indirect and less immediate benefits of collaboration. This article contributes to the growing body of research on interdisciplinary collaboration by applying a distinct theoretical and analytical framework to emphasise the potential of grassroots collaboration and the role of power and influence on research within universities.

## Introduction

Often disciplinary and epistemological differences, combined with habitual ways of working in universities, reinforce traditional approaches to research (Cummings & Kiesler, 2008; Fitzgerald et al., 2020). As a result, assumptions about learning, research paradigms and methods become entrenched, with lived experiences often reinforcing personal and collective beliefs and practices in academia (van Manen, 1997). Traditional entrenched research approaches tend to be one-dimensional, which enable deep analysis of particular specific problems; however, increasingly complex socio-economic problems (for example, the 2008 Global Financial Crisis and the 2020 COVID-19 pandemic) require the wider lens analysis which interdisciplinary research provides as a better research vehicle (Brown, 2018; Wong et al., 2014).

The aim of this article is to examine one attempt at ‘breaking out’ of this traditional academic milieu and in the process reveal effective strategies and practices, while identifying challenges that emerge. This is an under-researched multi-layered complex process with implications for building interdisciplinary research in universities, which is often rhetorically spoken about by university administrations, but with limited efforts to make this a reality. Leahey notes that “… in many academic institutions, statements of support [for interdisciplinary research] from the administration are still decoupled from actual practice” (2018, p. S64). This decoupling in interdisciplinarity is reported by Shaw and Wiener (2000). It is also the subject of inquiry by Jürgen Habermas who recognised the functional importance of law and its ability to create fragmented and separate social and political spheres that lack an interdisciplinary legal-political science approach. This work has inspired the approach adopted in the research presented in this article. Specifically, the Habermas (1987) theory of societal action enabled analysis of the structural and social dynamics that facilitate or inhibit interdisciplinary research. By applying Habermas’ theory to a living example of collaboration within a tertiary institution, this article contributes a new approach to research on the area of interdisciplinary practices.

Through participating in interdisciplinary research groups, the status-quo noted by Habermas is challenged and researchers are prompted to revisit assumptions (Brookfield, 1995) and find new ways of working. This article focusses on one such challenge. It examines the initiation of a formative interdisciplinary Research Group at Federation University in Australia as “research active staff sharing a common research interest and past research performance that align with at least one of either the University’s Research Priority Areas or a School’s research priority themes” (quotation from internal university *Strategic Goals and Policy document*, 2020).

This article describes and analyses the establishment of the cross-school research group (CSRG) and as such has the purpose of providing insights into the process and the lived experiences—specifically, the journey of setting up and launching this group (Fitzgerald et al., 2020). Thus, the key research question that underpins this research is: What are the aspirations and expectations of staff involved in the interdisciplinary cross-school group, and how can they be supported?

To address the research question, the context of the formation of the CSRG is first described. Then, relevant literature on efforts to create interdisciplinary research groups within academia is critically reviewed to identify where this article aims to contribute to the existing literature. From this, the methodology section explains the qualitative analysis utilised in this research project, the conceptual framework, the method of thematic analysis and the data organisation process. Findings from this process are described, followed by discussion of the patterns and emerging themes to address the research question. The article concludes by indicating pathways for academia to break out of fragmented, conservative and often ineffectually administered interdisciplinarity, with implications for challenges that are identified.

## Research context

Federation University has a history of successful collaborations within the institution (intra-university), notably open access data projects between individual academics, librarians and an eResearch Centre (Steel et al., 2019), but not interdisciplinary research across specific Schools and disciplines. The concept of a cross-school research approach was initiated by the Deans from the three individual schools. During an initial meeting of staff from the three Schools initiated by the three Deans in November 2019, possibilities, synergies and potential collaborations for the research group were identified, together with an acknowledgement that the future focus of university research needed to be both multidisciplinary and interdisciplinary in nature. This initial all-staff three-School meeting provided an opportunity to discuss ideas to develop a proposal for a cross-school research centre. Feedback from staff surfaced commonalities in key research areas, consisting of policies, procedures and practices incorporating a wide variety of disciplines. A key focus during the discussion was the facilitation of grant applications, exploring new synergies and expectations related to publications, and achieving world standard Excellence for Research in Australia (ERA) levels. The open dialogue represented a process whereby the Deans were genuinely seeking engagement and collaboration with colleagues and were supportive of the emergent and flexible nature of grassroots collaboration across Schools. In recognition of the emergent and flexible nature of the process that had been initiated, the Deans et al. committed to the collaboration decided to build capacity via a cross-school research group as a transitional step towards a Research Centre. Subsequently, a Working Party comprising one member from each of the three Schools was established and an expression of interest in becoming an active member of the research group was extended to all staff in the three Schools. The working party then invited all staff to respond to a survey that aimed to determine the aspirations of the CSRG to establish foundational expectations (see further details in data and data collection and methodology sections, below).

### Review of interdisciplinary research literature

When conducting a critical review of the academic literature in the disciplines of education, business and health care, Aboelela et al. (2007) could not determine a clear definition of interdisciplinary research because it was open to interpretation, with many ways to interpret interdisciplinary research endeavours. Thus, this research team offered an expanded definition to aid funding agencies and formalised training:

Interdisciplinary research is any study or group of studies undertaken by scholars from two or more distinct scientific disciplines. The research is based upon a conceptual model that links or integrates theoretical frameworks from those disciplines, uses study design and methodology that is not limited to any one field, and requires the use of perspectives and skills of the involved disciplines throughout multiple phases of the research process. (Aboelela et al., 2007, p. 341)

This definition is adopted in this study as it provides a clear focus for researchers producing such research to address ‘real-world’ problems that demand a wide lens of analysis, not deep—often dense and impervious—exploration.

Since the early 1980s, higher education reforms across advanced economies have resulted in metrics-driven evidence-based research activities that highlight tangible economic benefits. Doidge et al. have identified that that such reforms work “against cross-institutional and interdisciplinary collaboration” (2020, p. 1130). Current directions in world-leading knowledge economies suggest that this approach remains narrowly one-dimensional and reliant on traditional Anglophone-dominated institutions. As Doidge et al. (2020) argue, there is “a missed opportunity” to engage with different knowledge economies, particularly within Asia, that foster a more rounded approach to interdisciplinary pedagogy and research.

Some recognition of the need to change the research agenda towards interdisciplinary research is evident. In the United Kingdom (UK), for example, after the 2008 Research Assessment Exercise (RAE), university-level research has been shifting its social contract for over a decade because ‘drilling down’ in research has not necessarily produced the wide lens of analysis referred to earlier. Brown (2018) asserts that national research systems like the original RAE and the research excellence framework (REF) set-up in the UK produced discrete research knowledge domains that were too one-dimensional to have sufficient social impact on the complex challenges that researchers now face (Wong et al., 2014). To foster the game-changing solutions that may be born from interdisciplinary collaborations, the RAE’s existing assessment panels for 15 cognate research domains were combined and reduced to four in 2014 (Brown, 2018). In this way, Brown (2018) argues “that interdisciplinary research will produce those game-changing radical innovations that will help to solve some of the major social and environmental issues we face” (p. S28). Simultaneously, research impact became a key aspect of the assessment to recognise and acknowledge that research should not always ‘drill down’ (deeper into one discipline) but rather ‘break out’ from academia and extend its reach into society, policy and procedures.

In Australia, this need for greater emphasis on interdisciplinary research has also been recognised. Specifically, in the education discipline, it was noted in the 2012 Presidential Address of Australian Association for Research that:

Interdisciplinarity is at the forefront of research innovation and compels us continually to consider our own disciplinary identity as well as those of ‘other’ disciplines. Such critical, reflective practice is necessary to ensure education’s place as a serious academic discipline, rather than merely a commodity for the world of practice. (Halse, 2013, p. 151)

This innovation-based argument is also presented by Harrison et al. (2013) who demonstrate that education has become an interdisciplinary field of research with theoretical conceptions, thus moving far beyond its original conception as a means of training teachers. Yet, as Harrison et al. note, traditional education training is still well entrenched as “staff with strong theoretical backgrounds were not always encouraged in programmes where it was considered that those who engaged with the theory were detached from the ‘real world’ of teaching” (p. 504), which is seen as purely practice-based.

More broadly, the Australian Research Council (ARC) includes a ‘Statement of Support for Interdisciplinary Research’ on its website (ARC, 2015) that acknowledges commitment to “fostering excellence in research that traverses or transcends disciplinary boundaries and which synthesise or integrate methods and knowledge from multiple disciplinary domains”. The ARC uses this commitment to allow interdisciplinary research projects under the National Competitive Grants Program (NCGP) it administers. However, there is no specific structural change to the grant process to facilitate commitment to interdisciplinary research. Instead, it links such research to established discipline-based ERA metrics, such that “interdisciplinary research is that which has been assigned two or more distinct field of research (FoR) codes across the two-digit level”.

Structural change to the conduct of research is required to address the needs of interdisciplinary research which is “both dynamic and rich” (Brown, 2018, p. S26). It is ‘dynamic’ in several ways in relation to the cognitive distance between disciplines, need for integration of such discipline cognition, insights of external stakeholder partnerships and broader-based research design. It is also ‘rich’ because it can lend itself to three broad classifications of research design: problem-orientated (e.g. communication in medicine), challenge-driven (e.g. safety-mask wearing) and fundamental knowledge (e.g. theory development). In all three classifications, a holistic or multi-layered approach can integrate knowledge, further understanding, and create radical innovations. However, to bring about change to the existing academic silos, Brown (2018) asserts that systems and processes must be designed to allow researchers to break from incremental, discipline-specific developments and create the synergies needed to make these radical innovations. Thereby, creating new challenges and opportunities for interdisciplinary research work.

One major structural challenge to interdisciplinary research is overcoming obstacles to collaboration within institutions, which means confronting taboos within disciplines (Calhoun & Wieviorka, 2019). We also contend that it overlooks the resource-rich cultural and social capital existing within the broad academia. Two approaches are possible for such change to occur. One is from the top-down by the administration of research, altering the parameters around how research is conducted to include strong incentives for interdisciplinary research to break down existing cultural and social barriers. The UK provides an example of this approach with all the institutional problems this entails; for details, see Brown (2018). Another approach is one that emerges from the bottom up: grassroots breaking out by academics with the support of their universities. In Australia, the rigid ERA discipline-based metrics by the ARC makes it imperative that the alternative grassroots breaking out occurs by academics themselves, as argued by Halse (2013).

## Challenges and opportunities of interdisciplinary research

Arguably, universities and school departments are competitors in the education market, but in the research market, there can be great opportunities and incentives to collaborate rather than compete with other schools within one university, and with other universities. Such collaboration at the academic staff level provides a strong pathway to ‘breaking out’.

Universities and schools are seeking ways to reconcile what might be seen as a discord between competition and collaboration in interdisciplinary research (Sutherland-Smith, 2013). Previously, boundaries to international research collaboration, such as geographical distance, may have proven too great a challenge, with limited uptake and success (Kiesler & Cummings, 2002; Kraut et al., 1988) but technological innovations, international cohesion and human mobility have increased opportunities. The opportunity exists to generate innovative and complementary research pathways across universities and schools. These opportunities are bountiful, bridging the systems, cultures and organisational boundaries of different schools and universities, but negotiating different layers of existing cultural and social academic norms in research behaviour is the major challenge (Baguley et al., 2021). Cognate research domains do not necessarily equate to similar connections in how institutions are organised, or in staff reward systems, discipline structures and work practices (Cummings & Kiesler, 2008; Metzger & Zare, 1999). Such challenges are recognised in a study on sustaining interdisciplinary education:

This study confirmed that establishing and sustaining interdisciplinary education was dependent on efforts to bridge and build connections across discipline boundaries. However, if the partnerships resulting from these efforts are to endure and be sustained, they need modes of boundary crossing governance that disentangle School-based institutional processes. (Hannon et al., 2018, p. 1437)

To build the required connections “across discipline boundaries”, consideration must also be given to the interpersonal and behavioural dimension of researchers. In a survey of 2090 respondents of Australian academics, collegial engagement is a dimension deemed necessary to produce effective research outputs (Bazeley, 2010). As a dimension, this is described as sharing knowledge and expertise that may be topic-based or methodology-based. Bazeley (2010) asserts that this sharing need not be in only a leadership role but a collegial role. Such collegiality requires an element of confidence to share “without feeling ‘precious’ about ‘giving away’ ideas or having one’s ideas critiqued” (Bazeley, 2010, p. 899). However, this willingness to share can become reciprocal learning, learning from those around you. Without collegiality, the research system can become self-serving and therefore limited in benefits to a system. Interdisciplinary research and broad-based communities of practice can be such systems that foster collegiality, collaborations and connections (Moss & Kubacki, 2007), thereby counteracting the competitive and isolating environment of research systems in many universities.

In summary, the challenges to interdisciplinary research collaboration emerging in different national environments are well identified in the literature. That being said, case study research on projects or centres endeavouring to ‘break out’ of institutional and disciplinary divisions is less prevalent. The remainder of the article examines our study of the CSRG as one case study to strengthen the empirical base of previous research. To add to earlier studies a distinct conceptual framework, we used Habermas’ theory of societal action.

## Methodology

In interdisciplinary research, where the overall objectives are problem-orientated, methodology tends to be less discipline-specific (Siebert et al., 2020) with new and adapted methodological tools emerging as a result. To allow for the possible emergence of unanticipated findings, the authors adopted general qualitative methodology with a conceptual framework to guide the data collection and thematic collation. The methodology presumes a focus on the meaning attributed to collaboration by staff and the objective of a better understanding of academic aspirations for engagement.

Qualitative approaches to research underpin this project (Ezzy, 2002). As such, interpretative methods of analysis of data were fit for purpose. The authors elected to utilise thematic analysis as the method best suited to the task of classifying, testing and then organising data on the reported experiences of social and professional life. As thematic analysis allows for a methodical approach to processing data, it became possible at key stages to reflect on the categories in a conceptual framework derived from Habermas and the unfolding research process. The thematic analysis of responses to open-ended questions goes to the heart of relations between researchers, which would in turn bring out rich data on the academic experiences in this at Federation university, provided that a methodical approach to interrogating the data was taken. In the data organisation section below, the authors describe the methodical approach taken, breaking down each step in the process.

**Fig. 1:**
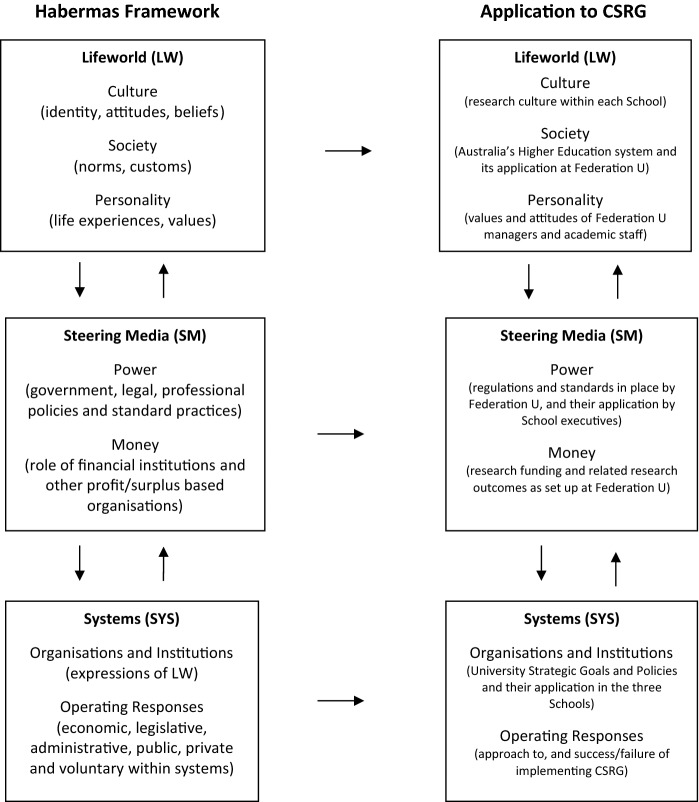
Habermas’ theory of communicative action and the CSRG. *Source* Adapted from Habermas (1987) and Zacharias (2007)

Qualitative methodology and thematic analysis were a fit for the study’s aims. The authors treated Habermas’ theory as a mechanism, a guide and a tool for organising the data from the interviews. This stage in the research process was built on awareness of other studies using Habermas’ framework. Two instances of research influenced by Habermas’ theory of communicative action that can be noted are evaluations of educational theory (McNiff, 2007) and COVID-19 research (Pulido et al., 2020). The authors also anticipated that this article would highlight a study of intra-university collaboration not previously addressed in this field of applied research and therefore contribute to this field of knowledge. With Habermas’ framework in mind, one member of the group summarised Habermas’ broad categories of Lifeworld, Steering Media and Systems into subcategories (see left-hand side of Fig. 1) and then constructed an interpretation of how they might apply to CSRG (see right-hand side of Fig. 1).

## Conceptual framework

The overarching Lifeworld (LW) consists of intangible interpretative elements made up of culture, society and personality. Culture and Society are terms evident from the descriptions in the top left-hand box of Fig. 1. Distinct national/global and cultural identities (whether it is country, state, region or even global capitalism) constitute the historically rich ‘Lifeworld’ which underpins the next two levels. The second level comprises ‘Steering Media’ (SM) which is critical in setting up how any organisation engages with the political, business and community development processes. SM consists of abstract actors reflecting the political and legal framework that sets laws, regulations and standards (Power) and the financial institutions and investors driven by profit/surplus (Money). The third level is ‘Systems’ (SYS). This refers to the institutional framework comprising the extant stakeholder organisations, institutions and socio-political economy of a country. SYS are organisations and institutions that are “expressions of the Lifeworld and which follow Lifeworld concerns” (Zacharias, 2007, p. 88). In all contexts, these are operating systems by which tangible organisations function in the community/local space in response to the SM. Essentially, SYS operate through actions of the SM but follow LW imperatives; thus, the first arrow points downwards. However, when SYS drift away from the LW, they create the critical need for institutionalised SM to recalibrate its approach whether to counter the SM drift or augment this drift (Zacharias, 2007, p. 91); in this way, SYS can influence institutions of power; thus, the second arrow points upwards across the three levels.

The three framework levels and their relationship to each other are represented on the left-hand side of Fig. 1 as exposited by Zacharias (2007) of the Habermas (1987) theory of societal action. The right-hand side is an interpretation of this Habermas framework as it applies to this study. Within each box on the CSRG application right side, each of the Habermas concepts apply to the way research exists and is conducted within Federation University and its Schools.

## Research/study participants

The participants in this study included the team leaders (3) and 15 academic staff representing the School A (5), School B (4) and School C (6); all three schools are in the broad Humanities, Arts and Social Science (HASS) fields. The four co-authors of this paper include the three team leaders and an academic from School B who was also a research participant. The project, ‘Breaking out: The genesis, development and implementation of an interdisciplinary university cross-school research group’ was approved by the Federation University Human Research Ethics Committee (Project: B20–121). This approval was on the basis that all data from participants are de-identified to ensure privacy of individual responses. Thus, all quoted responses in this paper are identified by a random number and School A, B or C.

## Data collection

The data for this research consisted of responses to a staff survey, together with team leader email communications. The survey was designed during face-to-face meetings and emails (once the COVID-19 pandemic brought about campus closure during 2020/2021). At the conclusion of the survey development process, a schedule of questions was crafted into a final survey. The first set of questions was on policy and practice: 1. What do you envisage for this group? 2. What does practice mean to you in your discipline? 3. Where are the gaps in policy and practice in your discipline? 4. What would be a good project for a grant in your area? and 5. Can you envisage connections with other schools? Staff were then prompted to rank the following in order of preference as principles of group engagement and aspiration and hopes for the CSRG—Social interaction; Preparing for and submitting grants; Provision of policy advice; Publications; Mentoring; and Other. The final survey question referred to the skills (organisational and social), experience (including grant development and application), theoretical and methodological skills and previous multidisciplinary team research experience.. Staff were requested to rank these skills/experiences in preferred order (see Appendix 1 for the complete survey). A total number of 14 survey responses were received from the three schools.

## Data organisation process

A select sample of the survey responses was trialled to test the validity and heuristic value of the categories and subcategories. A professional staff member from the university’s Research Services provided a random survey sample from each of the three participating Schools. Three of the authors each read a survey from the two Schools of which they were not members. This method assured validity. The three authors then met and compared observations.

After a considered discussion of the categories and the subcategories, a conclusion was reached that the broad categories (Lifeworld, Steering Media and Systems) from Habermas’ framework offered subcategories that we believed could be operationalised more effectively at a general level to ensure that the analysis would be effective. As a next step in our method, the three members who conducted the trial reviewed all of the surveys from the two schools that we were not members of. We applied the same methodical step from the trial to the entire survey and interview data set; this time applying the subcategories from Habermas’ framework and not those from the application to CSRG (see Fig. 1). As we proceeded through this stage, we coded. We then reconvened and discussed the coding experience and shared our individual findings. This process enabled a robust approach for ensuring triangulation and data credibility.

In the next section, we identify and discuss the five key findings organised as themes. These are as follows: (1) collegiality, collaboration and connections; (2) Are research grants a priority? (3) systems; (4) Publish or perish? ; and (5) role of emotions.

## Findings and discussion

### Collegiality, collaboration and connections

“I envision finding opportunities to connect with other research disciplines to conduct inter-connected research opportunities” (1C).

Increasingly, interdisciplinary research is gaining traction within universities, particularly in the education discipline (Newman et al., 2014). While this research supports the extant literature relating to the gaining of traction, it also reveals deeper insights about participants’ aspirations and motivations, an aspect of interdisciplinary work that is under-examined. One of the key questions underpinning this research was to identify the aspirations and expectations of staff involved in the interdisciplinary cross-school group. The survey results are predominantly linked to the Habermas Lifeworld (LW), represented as the creation of a cross-school research culture, and one that emerged specifically in response to the university context. Participants’ aspirations reflected an openness—“I’m therefore going in with an open mind” (3B) together with a desire to collaborate. For example, one participant stated: “Collaboration on new projects; mentoring of ECR [early career researchers] and MCRs [mid-career researchers] (provides) greater capacity. We can stand out in research together” (2A); while another claimed that “Genuine collegiality would be ideal” (3A). It was also anticipated that there would be opportunities for “collaboration on a variety of topics but overall cross-fertilisation of ideas and research opportunities” (2B). The respondents highlighted the attitudes and values of the academics who appreciated the opportunity to explore diverse approaches to research and examine disciplinary intersections.

While the literature reveals conduct and governance of research that focusses on cross-school collaborations and interdisciplinary research groups and centres (e.g. Metzger & Zare, 1999; Parker & Hackett, 2012), there is limited research that has focussed on participant assumptions and their emotions (Boix Mansilla et al., 2016). Through surfacing and making visible the underlying assumptions, aspirations and decisions researchers make to do educational work, we gain deeper insights into personal and professional motivations. In its broadest sense ‘educational work’ recognises that for any kind of learning to take place material/cultural infrastructure support is needed. It also requires thinking about pedagogy in its broadest sense as a relational encounter among individuals through which possibilities for intellectual growth are created (Zembylas, 2007). As Newman et al. also suggest, “Expanding our idea of educational work enables us to include all kinds of building and maintaining of human relationships” (2014, p. 327).

### Are research grants a priority?

Analysis of the data highlighted one noticeable observation between our expectations and the data. Despite the university setting a priority on securing grants and increasing research income (elements of SM), few staff in their responses to open-ended questions mentioned the potential role of the CSRG in securing grants. Our instinct was to check the final part of the survey to evaluate if there was a pattern of priorities emerging from closed questions. Survey questions five and six solicited priorities from colleagues. The schedule asked respondents to rank a given set of *priorities* in order of importance (question five) and then rank a given set of *skills* according to what they believed they could offer the CSRG (question six, see Appendix 1).

The rankings suggest that grants were important to many of our respondents. A majority ranked grants very highly (as a first or second priority in question five, eight out of 13). The responses were polarised with other respondents ranking them very low. When it came to skill sets (question six), three identified grant writing as the skill they brought to the group. More, however, ranked experience with multidisciplinary team research very highly (five out of 13 as a first or second skill in question six). This suggests a significant minority of respondents foresee teamwork as a strength they brought. All the other skills (theory, methodology and organisational skills) were similarly valued by a significant minority of staff (five out of 13) who completed the survey.

Therefore, there is a mismatch between the experiences of staff in the open-ended questions where grants are scarcely mentioned, yet according to the rankings, many colleagues attached high importance to securing grant funding. Our inference from the rankings of priorities is that grants are *implicitly* important to many staff as part of the traditional research culture. The subsequent grants rounds run by the CSRG is not incorporated into the research brief of this paper. However, as a brief note, the level of support from staff for the 2020/2021 CSRG grants round suggests that grants are indeed an active priority for academics seeking to collaborate in interdisciplinary research.

### Systems

Contrary to expectations, the coding exercise produced little by way of responses meriting codes in Steering Media, Power or Money (SMP, SMM). Comments that were coded SMM or SMP typically spoke to skill and priority rankings relating to grants (SMM) or research projects that intervene in business, public sector or educational settings with implications for power in those contexts (SMP). Our interview schedule did not explicitly solicit views on the operation of power and money in intermediating the school culture and managerial and financial systems of the university. Nevertheless, some respondents chose to share their experiences. Some examples included:

The focus suggestion (and title) is ‘Policy Systems and Practice’. This Group can support work being done in all Research Priority Areas [RPAs] and how the outcomes of these areas can be implemented (or how not) that institute better systems of operation that embed the technical outcomes from these other RPAs, and then how such systems are put into practice. If you use a social science framework as a basis for our work, the focus will be even better understood and could create great grant applications. (6B)

My interest lately has been in strengthening the evidence base for literacy pedagogy, in particular the vocabulary curriculum. The Australian Curriculum is vague on vocabulary and the benchmarks suggested for vocabulary within the literacy progressions do not have a clear evidence base. (2C)

The university’s research focus is on ‘Research Priority Areas’ and the ability to generate strong ERA outcomes in these areas. (6B)

Too much research focusses only at one level given the narrow discipline focus. For example, understanding of innovation and entrepreneurship from a narrow level gives you the economistic responses based on data that comes out of the Global Entrepreneurship Monitor (GEM) without much context to why it occurs that way…(6B)

Three of the four comments above are attributed to one respondent. As indicated, respondents related and referred only minimally to the two higher levels of Habermas’ framework and this may be because the survey did not explicitly ask about those areas. However, an alternative view is that respondents may see the CSRG as a ‘ground up’ initiative with the opportunity to move out of their discipline silos, and that the process would work through collaborating across the Lifeworld. In summary, collaborative research should be user generated.

### To publish or perish?

Publish or perish is often quoted as the guiding principle and driver of academic research (Costello, 2012; Davis et al., 2020; Nalbone, 2011; Yan & He, 2015). This principle has been constant over decades, and applies across locations and disciplines (Huang et al., 2006; Qiu, 2010). Reasons for this drive to publish research in high-ranked journals may be objective or subjective but significantly, research prowess measured by publishing is linked to a university’s reputation (Linton et al., 2011). Further, co-authored publications generate higher rates of citation, particularly in the HASS fields (Haddow et al., 2017). The institution and respondents surveyed in our analysis are no exception to the ‘publish or perish’ mantra, and therefore may be expected to respond likewise, however, that was not a key focus of their responses herein. One respondent, however, referred to a previous experience in another university:

I envisage this group will be able to provide a space for greater communication and cooperative ventures across the university. I remember when [university] initially set up the [research network]. In the short time that it was operational, outputs included a number of small grants, a conference and a published book. As a member of this group, I learned a great deal from working with others from the Business, Arts, and Medicine faculties, in particular. I think there is a lot of potential for cross discipline research that could be supported within this environment. (4C)

Admittedly, responses to questions about what the respondents envisaged for the group included goals and objectives that may be perceived as paths to publishing, but publishing was not mentioned once by any of the respondents.

As previously discussed, the responses focussed on collegiality and mentoring, that is, on the relational aspect of the Lifeworld. As such, it may be perceived that academics are aware they must publish but also that there are different pathways to achieving this, and that they see cross-discipline collegiality and mentoring as a desirable and opportunistic pathway to this end.

### The role of emotions

In analysing our survey responses, it is also useful to identify and analyse the emotions expressed within the descriptions provided (Lamont, 2009). Such emotions are culturally embedded in the social identity of the group (Lamont, 2012). Emotions have a role to play in interdisciplinary collaborations because they can instigate and maintain creative work, and drive social movements (Parker & Hackett, 2012). Thagard and Kroon (2006) modelled cognitive consensus as developing alongside emotional consensus in a process where group members share both positive and negative views about the group objectives. Emerging from the Lifeworld, both positive and negative emotions can incite positive changes in processes and goals. However, negative emotions particularly can be triggered, for example, by disagreement, power imbalances, and overextension, which need to be managed to avoid hindering group success. Notably, our survey responses were overwhelmingly positive, such that comments that may be perceived as negative or criticism were given for the purpose of explaining a positive. The respondents suggested that: “It sounds as though the group will be quite large, which I expect will create a diverse range of expectations. I’m therefore going in with an open mind (3B). “Fantastic if the group could come up with a narrative and foster real connection between people, genuine collegiality would be ideal”. (3A).

Emotions are often researched and analysed as a concept distinct from others (e.g. cognition, systems, priorities) (Boix Mansilla et al., 2016). However, ultimately, it may be useful to acknowledge that the role of emotions in our respondents may interplay with the other four themes above and consider positive emotions are likely to have a ‘knock-on positive effect’ on the success of our collaborations.

## Summary of key findings

The key findings of the survey data suggest that participants envisaged an opportunity to collaborate in interdisciplinary research, within an environment that encouraged collegiality, creativity and promise. This reflects what Habermas has defined as the development of an academic culture, known as Lifeworld, and reflects the opportunities created within the specific university context. The values and attitudes were made apparent and indicated that there was a genuine willingness and openness to collaborate. Grants are implicitly important to many staff, yet participants did not select grant writing and grant success in response to the open-ended questions, but more so when prompted by specific, guiding questions related directly to the Steering Media (SM). Surprisingly, few respondents discussed or even mentioned the University, its structure or ecology (SMP), nor did they identify and highlight the ‘publish or perish’ mantra.

## Conclusion

This research study has highlighted individual assumptions and practices that so often become habituated and difficult to interrupt. From the responses by the participants, there was a strong desire for ‘genuine collegiality’, with the implicit view that currently the academic LW and its implementation by the university SM (both SMP and SMM) does not provide such collaborative collegial research work. Participants also expressed enthusiasm for the CSRG to work as a ‘ground up’ SYS initiative that enables them to extend outside their discipline silos, and in this way to build diverse approaches and human relationships. Thus, we concur with extant literature that suggests “the different academic disciplines within a single university appear to be separate communities of practice, each with its own values, beliefs and [academic] identity” (Wenger, 1998, p. 70). It became evident through our analysis of the initial data that there was an overwhelming desire and motivation to add to their current discipline focus by a unique opportunity to be an active member of a cross-school interdisciplinary research group. It was also evident that academics aspired to collaborate in a collegial and focussed group to advance research that has still an ERA ranking, but with elements of interdisciplinarity. This tension between the desire to collaborate across disciplines and adhere to traditional disciplinary research metrics like ERA has two implications.

The first implication is that the cultivation of an ecology of interdisciplinary collaboration requires constant, and renewal of, effort in the face of a dominant traditional disciplinary-based research culture. As Fitzgerald et al. note, an enabling and productive relationship “is challenging to develop, fragile to maintain and requires constant monitoring and nurturing” (2020, p. 6). For us, this points to future research activities that include questions and insights into the ways in which the research teams within this group were generated; how the academic identity of the individual researcher/s developed and the ways in which tensions were addressed. This research has identified and highlighted the aspirations and motivations as to why individuals and teams in higher education want to collaborate across disciplinary borders to enable cross- disciplinary research and promote individual and collective learning. While the participants acknowledged that publications and grants were indeed a focus, they were not the driving factor for participating in the CSRG. The critical driver was the emotional value of deep social interaction that interdisciplinary collaborative research offers. The participants were very open to such positive emotions and the ‘passing-it-on’ collegial culture inculcated through the CSRG. From a Habermasian perspective not applied elsewhere in the relevant research on collaboration in universities, this driver provides a grassroots SYS alternative from the strong top-down power and influence of intransigent LW of academic culture that is discipline-based promotion and implemented through the SM of individual universities.

A further key implication of this study is that policies and systems require specific collaborative activities and outcomes with relation to research. The traditional academic discipline-based Lifeworld culture, which the participants were aware of in their responses, underpinned their choice to become involved. They saw the CSRG as a disruptive institutional-based opportunity to be innovative and creative in a lateral thinking approach, rather than their ‘normal’ vertical (deep) disciplined-based thinking approach. As Hannon et al. suggest: “While the literature on interdisciplinary education emphasises academic collaborations and leadership, there has been less attention to the role of institutional processes—mediated by procedures, artefacts and routines—in supporting and sustaining interdisciplinary education” (2018, p. 1424). Rather the CSRG offers a focus outside the one level provided by an often-narrow discipline. It offers interaction on complementary skill sets with staff across the three Schools; on skills like theoretical and methodological expertise; quantitative and qualitative approaches and analysis of data and sharing research expertise. In many ways this research raises further questions about the impacts and maintenance of research groups not often addressed in the literature on team research in universities. If universities are to develop policies and systems that better sustain collaborative research, then supporting the organic development of lively academic Lifeworld cultures is essential.

Overall, the research question set out to inquire as to the aspirations and expectations of staff who opted to become involved in the interdisciplinary CSRG, and how this research group can support such goals. This provides a Habermasian societal action point of focus to ‘break out’ of the traditional modes of educational research operation that overwhelmingly exist within Australian universities. The participants indicated a strong desire to engage with colleagues from diverse disciplinary backgrounds so as to ‘break out’ from existing academic silos, despite admitting uncertainty as to what it would involve. Expectations for strong emotional connection to colleagues with diverse skills and expertise are a counter-SYS approach to standard SM operations inbuilt into the LW of the Australian academia. This study found that engagement that counters the discipline-based competitive and isolating academic environment is an important dimension of research performance and was described by participants in emotional terms: interest, involvement, commitment, persistence. Findings such as this highlight the challenge and the importance of producing research output through a collegial system that allows researchers’ positive emotions and a ‘passing-it-on’ culture in order to build connections and flourish. Such a collegial system is counter to the current LW and SM top-down competitive publishing system and is represented in Fig. 1 by arrows pointing upwards from SYS through to changing the LW. How to build such a collegial system to support a bottom-up strategy like CSRG is indeed a challenge recognised by the responses in this study.

Acknowledgement must be afforded to the three School Deans and their initiation of the idea of establishing and funding interdisciplinary research in the humanities and social sciences, business and education. The Deans listened to their staff and recognised an unfulfilled desire to broaden the academic collegial system that has increasingly become narrower as pointed out by Doidge et al. (2020). Such support is critical going into the future in order for the expectations of these participants to be realised. The ability of Deans to strongly support the clearly identified impetus for such a collegial system will be of interest and critical to the future success of the CSRG. It is through the researchers working successfully in the CSRG and signalling to the Deans that this interdisciplinary work is credible which will provide the impetus for SM to change, with SMM (funding) and SMP (strategies) altered to reflect a more bottom-up LW academia.

Using Habermas as a guiding conceptual framework to organise and code the qualitative data unveiled the complex and multi-layered nature of cross-disciplinary research. Connecting theory to informing a framework for data organisation, the Habermasian framework became a valuable analytical tool not used elsewhere in the literature on barriers and enablers to interdisciplinary research or applied studies of research collaborations. However, the methodological approach and resulting analysis are also replicable in other contexts of cross-disciplinary collaboration making this methodological adaptation rigorous, durable and transferable.

This research has highlighted key understandings about the nature of interdisciplinary work in universities that often remains implicit and under-examined. There are four key understandings that have emerged from this limited, but revealing study:

1. institutionally, there is *value* in interdisciplinary research;
2. documenting and examining the *process* of establishing a cross-school research group and gathering and analysing data related to the process was critical as it allowed a deeper knowledge about individual and collective perspectives and aspirations, thus working from the ‘ground up’;
3. the Habermasian theoretical framework provided an *analytical tool*, enabling the identification of the importance of the academic Lifeworld and the role of institutional power and influence on research, and;
4. *institutional support* is essential to success so that the Steering Media can be more encouraging to the interdisciplinary work of academics.

## Cross-School Research Group: the next phase

The efforts to enact policy and build on the institutional research culture continue. Rather than institutional and cross-school support being “decoupled from practice” (Leahey, 2018), the progression of the aims and objectives of the group have maintained a forward momentum. The CSRG Leaders developed an internal, competitive funding round following the Deans’ and individual Research Committee approval and have financially supported four interdisciplinary research projects, representing a collective of 20 academic staff from the university and external institutions. It is anticipated that the cross-school research projects will lead to quality publishable outcomes (in priority ERA codes) and have the possibility of securing further external funding, which ultimately will lead to the establishment of a university research centre.

### Practice what you preach

Establishing the cross-school research group (CSRG) enabled the authors, as a collective of individuals with clear and often longstanding research outcomes based on individual disciplines, to articulate a new philosophy based on our university context. The ‘invitational tone’ sent as an Expression of Interest was designed to encourage staff from all disciplines to participate and clearly identify the need for representation across all career stages. A key aim was to “support and progress the development of a Cross-School research culture…[whereby] through a joint team effort, we aim to maximise our potential for success in research*…*We need your ideas, skills, and drive!” (EOI, April 2020).

Grant submissions to CSRG required the following: at least one ECR per submission, a representative from each of the three schools (A, B and C), new research collaborations/networks; projects that show a capacity to secure further external funding in the future, identification of impact of the research, ongoing staff only and one application per staff member. Reflecting on progress to date, the establishment process has highlighted the Habermas contention that a foundational approach to interdisciplinary research working from the ‘ground up’ will indeed promote a move towards a multi-dimensional and outward focussed research practice. As stated, a key overall finding from this research has been the contribution to an appreciation of a deeper and more bottom-up Lifeworld and the research culture that can be nurtured and sustained in such interdisciplinary work. While there was a somewhat surprising lack of reference to systems and structures, it is imperative that systems and structures are known, acknowledged and managed. As Newman et al. suggest: “In its broadest sense ‘educational work’ recognises that for any kind of learning to take place requires material/cultural infrastructure support” (2014, p. 323).

## Future directions

This research has raised questions worthy of further consideration and examination. For example, in what ways can a cross-research group help achieve better ERA levels? A key criterion identified as an outcome in the grant application and submission stipulated the submission of publications to high-quality journals together with targeting ERA codes, which is the focus of the traditional academic Lifeworld. A tension arises when determining the allocation of an article to a specific code when there are interdisciplinary colleagues with individual focus in different codes. Further questions for the next phase of the research include: How did your research team deal with the tensions inherent in allocating ERA research codes across competing disciplinary options? How did you connect and create your research team? What are your ongoing plans as an outcome of this research? and What should the CSRG do next? It will be during this phase that we will continue to monitor the impact of the research and the sustainability of the research culture and the contribution to the academic Lifeworld, published outputs and external grant success.

## Appendix 1: Cross-School Research Focus Group: survey questions April 2020

**Working title: policy and practice**

- What do you envisage for this group?
- What does practice mean to you in your discipline?
- Where are the gaps in policy and practice in your discipline?
- What would a good project for a grant in your area? Can you envisage connections with other schools?

**Rank the following in importance for you as a member of this group**

- Social interaction
- Preparing for and submitting grants
- Provision of policy advice
- Publications
- Mentoring
- Other

**What would you hope to bring to the group? Rank the following in order of importance**

- Organisational and social skills
- Experience in sourcing grants and crafting applications
- Expertise in theory
- Skills in methodology
- Experience with multidisciplinary team research
